# Assessment of the effect of betaine on *p16 *and *c-myc *DNA methylation and mRNA expression in a chemical induced rat liver cancer model

**DOI:** 10.1186/1471-2407-9-261

**Published:** 2009-07-30

**Authors:** Yan-ping Du, Jun-sheng Peng, Ai Sun, Zhi-hong Tang, Wen-hua Ling, Hui-lian Zhu

**Affiliations:** 1 School of public health, Sun Yat-Sen University, 74th Zhongshan Road II, Guangzhou 510080, PR China; 2 The 6th Affiliated Hospital and Gastrointestinal Disease Institution, Sun Yat-Sen University, 26th Ruancun across-Road II, Guangzhou 510655, PR China; 3 Guangzhou health education institute, 92th Huijing Road, Guangzhou 510403, PR China

## Abstract

**Background:**

The development and progression of liver cancer may involve abnormal changes in DNA methylation, which lead to the activation of certain proto-oncogenes, such as *c-myc*, as well as the inactivation of certain tumor suppressors, such as *p16*. Betaine, as an active methyl-donor, maintains normal DNA methylation patterns. However, there are few investigations on the protective effect of betaine in hepatocarcinogenesis.

**Methods:**

Four groups of rats were given diethylinitrosamine (DEN) and fed with AIN-93G diets supplemented with 0, 10, 20 or 40 g betaine/kg (model, 1%, 2%, and 4% betaine, respectively), while the control group, received no DEN, fed with AIN-93G diet. Eight or 15 weeks later, the expression of *p16 *and *c-myc *mRNA was examined by Real-time PCR (Q-PCR). The DNA methylation status within the *p16 *and *c-myc *promoter was analyzed using methylation-specific PCR.

**Results:**

Compared with the model group, numbers and areas of glutathione S-transferase placental form (GST-p)-positive foci were decreased in the livers of the rats treated with betaine (*P < 0.05*). Although the frequency of *p16 *promoter methylation in livers of the four DEN-fed groups appeared to increase, there is no difference among these groups after 8 or 15 weeks (*P > 0.05*). Betaine supplementation attenuated the down-regulation of *p16 *and inhibited the up-regulation of *c-myc *induced by DEN in a dose-dependent manner (*P *< 0.01). Meanwhile, increases in levels of malondialdehyde (MDA) and glutathione S-transferase (GST) in model, 2% and 4% betaine groups were observed (*P < 0.05*). Finally, enhanced antioxidative capacity (T-AOC) was observed in both the 2% and 4% betaine groups.

**Conclusion:**

Our data suggest that betaine attenuates DEN-induced damage in rat liver and reverses DEN-induced changes in mRNA levels.

## Background

Liver cancer is one of the most common types of malignancy, especially in Asia and Africa, and it is the second most common cause of cancer death in China [1,2]. Because of the very poor prognosis, the number of death remains stubbornly high [1]. Despite the effort in advancing the treatment of this disease in recent decades, long-term therapeutic outcome in liver cancer remains poor, and the prognosis is dire. Thus, prevention appears to be the best strategy for reducing the current prevalence of the disease [3].

The development of liver cancer is a multistep process of genetic alterations, involving the activation of proto-oncogenes and the inactivation of tumor suppressors [4-6], which lead to a continuous increase of uncontrolled cellular proliferation. Therefore, abnormal expression of related genes plays a crucial role in carcinogenesis. The *c-myc *proto-oncogene encodes a transcription factor involved in regulating cell proliferation, differentiation, and apoptosis [5,7-9]. A few studies have shown that hypomethylation of the *c-myc *gene promoter results in its over-expression, especially in the methyl-absent conditions [10,11]. *C-myc *over-expression is associated with liver cancer development by causing inappropriate gene expression which results in autonomous cellular proliferation [8,9,12]. The *p16 *tumor suppressor is an inhibitor of cyclin D-dependent protein kinases, and it halts the cell cycle in the G1 phase [13]. Several recent studies have revealed that extensive DNA methylation is the primary cause of *p16 *inactivation in liver cancer patients, and that hypermethylation of *p16 *occurs not only in the liver cancer but also in the pre-neoplastic liver tissue as well [14-16]. Abnormal changes in DNA methylation and mRNA expression of *c-myc *and *p16 *are, therefore, the two important stimulatory factors in the development of liver cancer [14-16].

There is a growing body of evidence which supports that chemoprevention is the cornerstone for precluding liver cancer [3,17]. Betaine, found particularly in wheat, spinach, and sugar beets, is an active methyl-donor. The main biochemical function of betaine is to transfer one-carbon moieties to maintain normal DNA methylation pattern in the body [18-21]. Thus, it is reasonable to propose that betaine may help to prevent liver cancer through regulating the expression of proto-oncogenes and tumor suppressors by stabling their mythylation patterns. Although some studies have suggested the role of betaine in preventing alcoholic liver diseases [18,20], the effect of betaine supplementation in preventing liver cancer has not yet to be investigated. The purpose of this study is to determine whether betaine supplementation attenuates the development of liver cancer in a well established rat model induced by diethylinitrosamine (DEN) treatment [3,17], as well as the exploration of mechanisms underlying the attenuation effect.

## Methods

### Animals and dietary intervention

Seventy pathogen-free, weanling male Sprague-Dawley rats (150 ± 10 g) were purchased from the Animal Center of Guangdong Province, and were kept in metabolism cages with controlled temperature (23 ± 2°C) and humidity (55% ± 5). All rats were acclimated for one week on a standard AIN-93G diet, supplying 187 g/kg protein, 70 g/kg fat, 50 g/kg fiber, and 3970 kcal/kg energy [22]. After a week of acclimatization, rats were divided into five groups according to their body weights. The control group was provided with distilled water and AIN-93G diet. The four experimental groups were given 0.01% diethylinitrosamine solution (DEN; Sigma Chemical Co., St. Louis, MO) and fed AIN-93G diets supplemented with 0, 10, 20, or 40 g betaine/kg. They were designated as the model, 1%, 2%, and 4% betaine group, respectively. Rats were housed individually and had free access to food and water throughout the 15-week period. Body weight and food consumption were recorded weekly. Betaine was purchased from Cultor Ltd. (Finnsugar Bioproducts, Finland). All the animal work procedures were approved by the Institutional Animal Care and Use Committee of Sun Yat-sen University.

### Assay of serum marker enzymes

Five rats from each group were sacrificed by exsanguination under anesthesia after receiving the experiment diet for 8 or 15 weeks. Blood was collected by femoral artery puncture. Serum levels of alanine aminotransferase (ALT), aspartate aminotransferase (AST), alkaline phosphatase (ALP) and Gamma glutamyl transferase (GGT) were measured by HITACHI autoanalyzer according to the standard procedures using commercially available diagnostic kits (Sigma).

### Assay of hepatic antioxidase and lipid peroxidation

The liver tissue were rapidly removed, washed in 0.9% NaCl, kept on ice, and homogenized in ice cold isotonic Na chloride. After centrifugation, the supernatant was collected. Levels of hepatic antioxidase and lipid peroxidation, including MDA, SOD, GSH, GST, and T-AOC, were measured using corresponding diagnostic kits (Nanjing Jiancheng Bioengineering Institute, Jiangsu province, China). Protein concentrations were determined by Coomassie Blue Staining method (Nanjing Jiancheng Bioengineering Institute, Jiangsu province, China).

### Histopathology and immunohistochemistry

The livers were removed and 3 slices were taken from each sublobe and fixed in 10% buffered formalin for histopathological examination. Two sections from each slice embedded in paraffin were prepared for the histopathological and immunohistochemical examinations. One section was stained with hematoxylin and eosin (H&E) and was examined under light microscopy for diagnosing liver lesions. Another section was used for Immunohistochemical staining of GST-p. The deparaffinized sections were incubated with a rabbit polyclonal anti-GST-p antibody (1:1,000 dilution, MBL, Nagoya, Japan) overnight in a humidified chamber at 4°C. The sections were then washed with PBS, and incubated with a correspondent secondary antibody. Subsequently, the sections were reacted with diaminobenzidine and hydrogen peroxide for 10 min. Control sections, in which the primary antibody was omitted, were treated in the same way and showed no immunostaining. Slides were examined by light microscopy, and the numbers and areas of GST-p-positive foci (>0.2 mm diameter) and the total areas of the liver sections were quantified using Scion Image (Scion Corp., Frederick, MD, USA).

### Methylation-specific PCR (MSP)

Genomic DNA was extracted from frozen tissues using a DNeasy tissue kit (Promega, Madison, WI, America) following the manufacturer's instructions. Methylation status of the CpG islands in the promoter region of *c-myc and p16 *was determined by bisulfite modification and subsequent methylation-specific PCR, as previously described with some modifications [12]. Briefly, 2 μg of genomic DNA was used for bisulfite modification. MSP was performed using methyl-specific primers (Forward and reverse primer for *c-myc were *5'-AAA CGA TAA GAG GCG GAT ATA TAA C-3' and 5'-ATT TTC CAA CTC AAA AAT CTA ATC G-3'. Forward and reverse primer for P16 were 5'-TAG TAT TGT ATT AGG TAG GGG CGC-3' and 5'-TAT CGA TAA CCC GAA AAA CGT T-3'.) and unmethyl-specific primers (U forward and reverse for *c-myc were *5'-ATG ATA AGA GGT GGA TAT ATA ATG T-3' and 5'-TTT TCC AAC TCA AAA ATC TAA TCA C-3'. U forward and reverse for P16 *were *5'-AGT ATT GTA TTA GGT AGG GGT GTG G-3' and 5'-ACC TAT CAA TAA CCC AAA AAA CAT T-3'). The PCR conditions were as follows: one cycle at 94°C for 5 min, 40 cycles at 94°C for 30 s, an annealing step for 30 s at the appropriate temperature (59.5°C and 53.0°C for primers to methylated and unmethylated *c-myc*, respectively, and 62.0°C and 60.0°C for primers to methylated and unmethylated *p16*), extension at 72°C for 30 s, and a final extension step at 72°C for 10 min. Final PCR products were separated by electrophoresis in a 2% agarose gel and visualized under UV illumination.

### Real time PCR (Q-PCR) for c-myc and p16

Total RNA was extracted from liver tissues using RNAiso Reagent (Takara Bio Co., Ltd., Kyoto, Japan). Complementary DNA (cDNA) was synthesized from 1 μg of purified total RNA using the first strand cDNA synthesis Kit (ToYoBo, Osaka, Japan). The *c-myc *and *p16 *genes were co-amplified with a fragment of the glyceraldehyde 3-phosphate dehydrogenase (GAPDH) gene, which served as an internal standard. Primer sequences for the *p16 *gene (GenBank accession L81167) were: 5'-TGC AGA TAG ACT AGC CAG GGC-3' (Forward Primer) and 5'-CTC GCA GTT CGA ATC TGC AC-3' (Reverse Primer). Primer sequences for the *c-myc *gene (GenBank accession AY679730) were 5'-GCT CTC CGT CCT ATG TTG CG-3' (Forward Primer) and 5'-TCG GAG ACC AGT TTG GCA G-3' (Reverse Primer). Primers for the GAPDH gene (GenBank accession AF106860) were 5'-ACC AAC TGC TTA GCC CCC C-3' (Forward Primer) and 5'-GCA TGT CAG ATC CAC AAC GG-3' (Reverse Primer). Specification of each pair of primers was tested by randomly sequencing three clones, and further confirmed by the melting curve analysis using Q-PCR [23]. The amplification efficiency of each pair of primers was tested by constructing corresponding plasmid. Only primers with similar amplification efficiency were used in this experiment. Q-PCR was conducted by amplifying 1.0 μl of diluted cDNA with the SYBR Green Real-time PCR Master Mix kit (TOYOBO Company, Japan) on the ABI 7500 sequence detection system (Perkin-Elmer/PE Applied Biosystems). The cycling conditions of forty cycles of PCR were 94°C/20 s, 58°C/20 s, 72°C/20 s. The amount of specific mRNA was quantified by determining the point at which the fluorescence accumulation entered the exponential phase (Ct), and the Ct ratio of the target gene to GAPDH was calculated for each sample. Each sample was run in triplet repeat and all the PCR data were analyzed with the ABI 7500 system software 4.0 version.

### Statistical analysis

All mean values are reported as the mean ± SD. Data were analyzed using a one-way analysis of variance, followed by least significant difference (LSD) test. Chi Square test and Fisher's exact test were used to compare the incidence of irregular DNA methylation. The level of significance was set at *P < 0.05 *in all cases. All statistical analyses were performed using SPSS software (Version 11.0) (SPSS Inc, Chicago, IL).

## Results

### General observations

Rats in the control group grew well throughout the entire experiment period. Although rats in other four groups showed fluffy fur, glassy eyes and decreased movement, the food intake and weight gain showed no significant difference among the groups (data not shown). Two rats of the 2% betaine group died during the 4^th ^week. In the 4% betaine group, one rat died during the 5^th ^week and another one during the 6^th ^week. One rat died in the 1% betaine group during the 15^th ^week.

### Histopathological Changes and immunohistochemical expression of GST-P in Rat Livers

**Figure 1:**
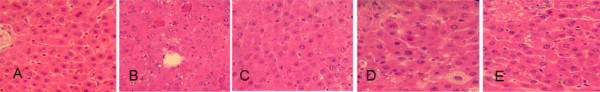
**Histopathological changes in rat livers**. Livers of rats in model, 1%, 2%, and 4% betaine groups: widespread malignant cells were observed, the model group showed most severe malignancy. Original magnification: 10 × 40. A: Control; B: Model; C: 1% betaine; D: 2% betaine; E: 4% betaine.

In the 8^th ^week, apomorphosis, necrosis, and accrementition were apparent in the liver slices of the model, 1%, 2%, and 4% betaine groups (date not shown). In the 15^th ^week, widespread malignant cells were observed in the four groups receiving DEN, whereas the model group showed most severely malignancy (Fig. 1).

**Figure 2:**
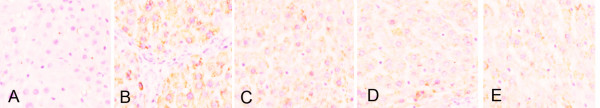
**Immunohistochemical staining of GST-p**. Control liver tissue: with weakly positive GST-p; Model liver tissue: with strongly positive GST-p expression. 1%, 2%, and 4%betaine groups showed liver tissues with positive GST-p expression. Original magnification: 10 × 40. A: Control; B: Model; C: 1% betaine; D: 2% betaine; E: 4% betaine.

**Figure 3:**
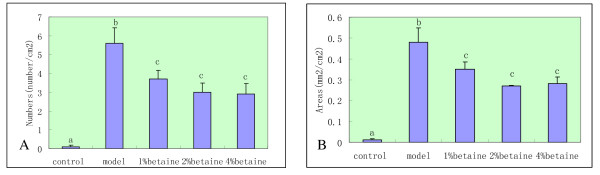
**The numbers and areas of GST-p-positive foci in different groups of rat livers**. Data are means ± SD. Different letter (such as "a" versus "b") on the top of each bar indicates *P *< 0.05 between them. A: Numbers; B: Areas.

Since the abnormalities in the expression of GST-p, a cell proliferation biomarker, play a critical role in the development of liver cancer, we assessed the expression of GST-p in liver cells. Numbers and areas of GST-p-positive foci in the mode group were significantly increased when compared with the control group (Fig. 2). Although there was not statistical difference among the four betaine-treated groups (*P *> 0.05), betaine supplementation was shown to decrease numbers and areas of GST-p-positive foci in the developing of liver cancer induced by DEN (*P < 0.05*) (Fig. 3). These observations collectively suggest that betaine decreases the process of hepatocellular carcinoma in rats induced by DEN.

### Contents of ALT, AST, ALP, GGT

We observed marked increases in the levels of ALT, AST, ALP and GGT in the four groups received DEN (*P < 0.001*). However, AST and ALT levels after 15 weeks in the 1% betaine group were shown to be deceased compared with that in the model group (*P < 0.05*) (Table 1).

**Table 1:** Levels Of ALT, AST, ALT and GGT

Week	group	ALT(U/L)	AST(U/L)	ALP(U/L)	GGT(U/L)
8 w	control	24.00 ± 2.12	197.60 ± 12.09	120.80 ± 10.47	7.40 ± 1.58
	model	37.50 ± 1.91*	247.00 ± 15.16*	219.50 ± 22.28*	27.10 ± 7.25*
	1%betaine	30.80 ± 4.32	196.00 ± 11.87^▲^	210.60 ± 19.60*	23.30 ± 9.90*
	2%betaine	38.20 ± 3.44*	214.40 ± 5.98*^▲^	216.60 ± 30.01*	38.10 ± 11.21*
	4%betaine	32.20 ± 4.91*	206.00 ± 7.78^▲^	234.00 ± 27.51*	14.80 ± 7.94
15 w	control	28.50 ± 3.08	215.17 ± 17.88	126.67 ± 15.86	11.42 ± 5.92
	model	60.14 ± 11.47*	291.00 ± 24.48*	232.86 ± 14.33*	41.57 ± 14.38*
	1%betaine	41.33 ± 4.55^*▲^	235.33 ± 19.12^▲^	233.67 ± 19.15*	32.67 ± 13.74*
	2%betaine	51.14 ± 9.80*	248.43 ± 19.03^*▲^	254.33 ± 41.02*	43.29 ± 8.59*
	4%betaine	57.57 ± 7.99*	253.00 ± 22.53^*▲^	246.17 ± 25.09*	42.50 ± 10.75*

*Compared with the control group, *p *< 0.05. ^▲^Compared with the model group, *p < 0.05*.

### Hepatic Antioxidase and Lipid Peroxidation

After 8 week, both MDA and GST levels increased in the model, 2% and 4% betaine groups (*P < 0.05*), but decreased in the 1% betaine group when compared with the model group (*P < 0.05*). T-AOC levels were also shown to be increased in the 2% and 4% betaine groups (*P < 0.05*) (Table 2) after 8 week.

**Table 2:** Hepatic antioxidase and lipid peroxidation

week		GSH mgGSH/gprot	GST U/mgprot	MDA nmol/mgprot	SOD U/mgprot	T-AOC U/mgprot
8 w	control	35.77 ± 3.39	27.37 ± 2.63	0.166 ± 0.055	170.09 ± 33.93	0.269 ± 0.089
	model	42.45 ± 6.87	38.62 ± 2.55*	0.357 ± 0.152*	164.98 ± 56.91	0.609 ± 0.248
	1%betaine	39.05 ± 6.77	31.36 ± 3.10	0.145 ± 0.068^▲^	190.86 ± 55.32	0.516 ± 0.201
	2%betaine	41.23 ± 2.59	35.14 ± 3.29*	0.361 ± 0.116*	178.75 ± 37.26	1.152 ± 0.407^*▲^
	4%betaine	41.34 ± 5.21	35.23 ± 4.22*	0.334 ± 0.124*	183.74 ± 83.19	0.755 ± 0.361^*▲^
15 w	control	38.21 ± 6.47	27.79 ± 6.73	0.236 ± 0.088	167.70 ± 44.01	0.363 ± 0.096
	model	38.22 ± 9.11	29.29 ± 4.23	0.299 ± 0.087	150.44 ± 57.59	0.912 ± 0.290
	1%betaine	40.19 ± 4.96	26.41 ± 3.46	0.222 ± 0.032	161.79 ± 40.13	0.564 ± 0.184
	2%betaine	39.99 ± 6.07	32.04 ± 6.89	0.231 ± 0.062	184.92 ± 38.38	0.942 ± 0.183
	4%betaine	36.13 ± 4.29	25.99 ± 5.07	0.307 ± 0.076	152.49 ± 45.54	0.777 ± 0.184

*Compared with the control group, *p *< 0.05; ^▲^Compared with the model group, *p < 0.05*.

### DNA methylation status of *c-myc*

**Figure 4:**
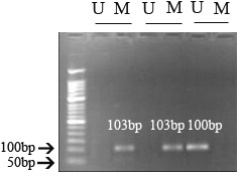
**DNA methylation status of the *c-myc *promoter region**. U: unmethylated, 103 bp; M: methylated, 100 bp.

All rats of each group were evaluated for aberrant promoter methylation of the *c-myc *gene using MSP. Methylated and unmethylated *c-myc *alleles were shown in Fig 4. There was no statistically significant difference among the groups after 8 (*P > 0.05*) and 15 weeks (*P > 0.05*) (Table 3).

**Table 3:** The methylation status of *c-myc *in the 8^th ^and 15^th ^week

group	8 w (M/U)	15 w (M/U)
control	5(0)	9(0)
model	4(1)	8(1)
1%betaine	5(0)	7(1)
2%betaine	4(1)	7(0)
4%betaine	5(0)	6(1)

M: methylated; U:unmethylated. The methylation status of *c-myc *in each group didn't show statistically significant differences after 8 weeks *(P > 0.05) *and 15 weeks *(P > 0.05)*.

### *P16 *Methylation

**Figure 5:**
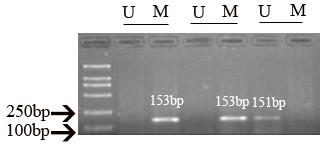
**DNA methylation status of the *p16 *gene promoter region**. U: unmethylated, 151 bp; M: methylated, 153 bp.

Fig 5 shows the results of *p16 *promoter DNA methylation by MSP. Although the frequency of promoter methylation for *p16 *in livers of the four DEN-fed groups appeared to increase, we didn't see a difference among these four groups after 8 or 15 weeks (*P > 0.05*) (Table 4).

**Table 4:** The methylation status of *p16 *in the 8^th ^and 15^th ^week

group	8 w (M/U)	15 w (M/U)
control	1(4)	3(6)
model	4(1)	7(2)
1%betaine	3(2)	6(2)
2%betaine	3(2)	6(1)
4%betaine	4(1)	7(0)

M: methylated; U:unmethylated. The methylation status of *p16 *in each group didn't show statistically significant differences after 8 weeks *(P > 0.05) *and 15 weeks *(P > 0.05)*.

### Effects of betaine on *c-myc *and *p16 *mRNA expression

**Figure 6:**
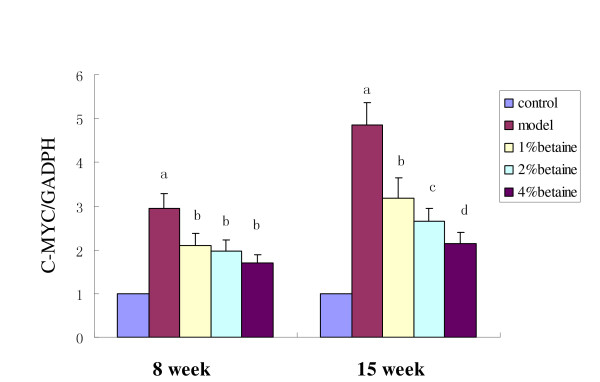
**Quantitation of mRNA levels of *c-myc *in the liver at week 8 and 15 in model, 1%, 2% and 4% betaine groups**. Results were expressed relative to the control group. Compared with the control group, the mRNA levels of *c-myc *in the model group were increased after 8 weeks (*P *< 0.001) and 15 weeks (*P *< 0.001). Betaine significantly decreased the up-regulation of *c-myc *expression induced by DEN in a concentration-dependent manner (*P *< 0.01). Data are means ± SD. Different letter (such as "a" versus "b") on the top of each bar indicates *P *< 0.05 between them.

**Figure 7:**
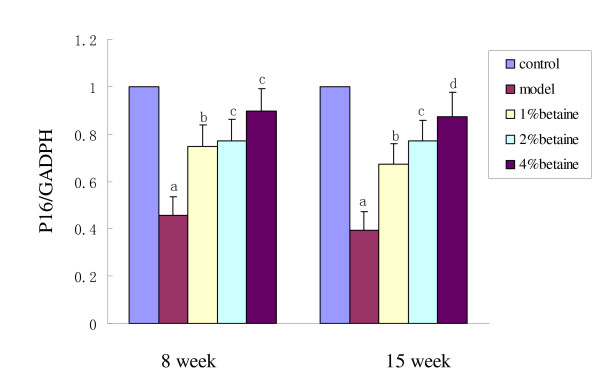
**Quantitation of mRNA levels of *p16 *in livers at week 8 and 15 in model, 1%, 2% and 4% betaine groups**. Results were expressed relative to the control group. The mRNA levels of *p16 *in the model group were decreased after 8 weeks (*P *< 0.001) and 15 weeks (*P *< 0.001) and betaine increased the down-regulation induced by DEN in a dose-dependent manner (*P *< 0.01). Data are means ± SD. Different letter (such as "a" versus "b") on the top of each bar indicates *P *< 0.05 between them.

Using Q-PCR, gene-specific mRNA expression was quantified in the livers from rats taking DEN solution (model, 1%, 2%, and 4% of betaine) for 15 weeks and results were expressed relative to the number of GAPDH transcripts. The mRNA levels of *p16 *and *c-myc *in livers of the control group were set at 1.00, and mRNA expressions of the four experimental groups were evaluated by its relative ratio. As shown in Fig 6 and Fig 7, DEN treatment enhanced the expression of *c-myc *after both 8 and 15 weeks, while betaine supplementation decreased the stimulatory effect on *c-myc *expression by DEN in a dose-dependent manner after 15 weeks (*P *< 0.01). On the contrary, betaine supplementation enhanced the down-regulation of *p16 expression *induced by DEN in a dose-dependent manner (*P *< 0.01).

## Discussion

Diethylinitrosamine is a powerful hepatocarcinogen known to induce cancer in experimental animals [3,17]. In our study, all rats given DEN developed the liver cancer, demonstrating that Sprague-Dawley rat is a suitable model in studying DEN induced liver cancer. DEN treatment was shown to significantly increase both numbers and areas of GST-p-positive foci (model group), while betaine supplementation (in all the three experimental groups) attenuated the effect of DEN. Although there was no statistical difference among the betaine treatment groups, betaine supplementation decreased the increase of GST-p in the developing of liver cancer induced by DEN. Collectively, these observations suggested that betaine can decrease the process of hepatocellular carcinoma in rats induced by DEN.

There is a strong correlation between oxidative stress and the occurrence of liver cancer [24,25]. Enhanced lipid peroxidation and/or a dysregulated antioxidant system have been associated with liver cancer in both experimental animal models and in humans [24,25]. Betaine acts as a methyl donor by participating in the generation of methionine from homocysteine through the catalytic action of betaine-homocysteine methyltransferase. Previous studies have shown that betaine is effective at preventing a variety of toxic injuries to the liver, such as those induced by niacin, CCl_4_, and hyperosmolarity [26-28]. Betaine appears to confer this protection by reducing peroxidation in the liver [28]. In our study, we found that MDA and GST levels decreased in the 1% betaine group when compared with the model group. Although the increase in the dosage of betaine did not increase the antioxidation activity in a dose-dependent manner, our observations are consistent with the suggestion by Hayes *et al*. that betaine is safe and nontoxic [29]. Betaine metabolism occurs predominantly in the liver [20], and DEN is able to generate acute damages in the rat liver [30]. We found that T-AOC level was higher in the 2% and 4% betaine groups than that in either the control or the model group. These observations suggest that betaine may increase the antioxidation activity of the rat hepatocytes.

Carcinogenesis is a multi-stage process characterized by continuous changes in genotypes and phenotypes [4,5,7-14]. In the same way, hepatocarcinogenesis is a multistep process involving genetic and epigenetic alterations of various oncogenes, proto-oncogenes, and growth factors, as well as tumor suppressors [4,5,7-14]. DNA methylation is a fundamental epigenetic process that regulates not only gene transcription but also histone acetylation and chromosomal stability [11,13,14,31]. DNA methylation, primarily at the C5 position of cytosine, affects gene expression during many biological processes, such as differentiation, genomic imprinting, DNA mutation, and DNA repair [11,13,14,31].

Aberrant methylation of cytosine residues at CpG dinucleotides in DNA is one of the most common epigenetic changes observed in the development and progression of human cancers, including that of the liver [31]. Shen *et al*. have shown that environmental factors may influence the frequency and concomitant degree of hypermethylation in multiple genes in liver cancer [32]. They have also demonstrated that oxidative damage can directly affect DNA methylation status [32].

DNA methylation has been recognized as an important factor in the inactivation of tumor suppressors, such as *p16*, and in the activation of proto-oncogenes such as *c-myc*. DNA hypermethylation, usually occurring at CpG islands in promoters, is a major epigenetic mechanism in the silencing of gene expression [15,16]. Aberrant methylation of CpG islands in the promoter region of *p16 *is associated with transcriptional inactivation of *p16 *itself. Methylation at CpG islands may inhibit gene expression, either directly by interrupting the binding of transcription activators to the promoter, or indirectly through methyl-binding proteins (MBPs) [15,16,32]. MBPs bind preferentially to methylated DNA sequences of a promoter and thereby silence its transcription by either competing with transcription activators for the binding sites, or by promoting histone deacetylation and chromatin remodeling that prevent transcription factors from binding to the DNA [32]. C5 cytosine methylation at CpG sites not only significantly increases carcinogen-DNA adduct formation at CpG sites, but also affects carcinogen-DNA adduct formation at surrounding sequences [33].

The *p16 *gene encodes an inhibitor of cyclin D-dependent protein kinases. It reduces enzymatic activity of cyclin/cdk complexes, leading to aberrant phosphorylation of another tumor suppressor Rb, which, in turn, accelerates cell proliferation [34,35]. When hypermethylation occurs in the CpG islands within the 5' flanking region of *p16*, its transcription is inhibited, which is a common marker of the early carcinogenic event [34,35]. We found that compared with the rats in the control group, the rats in all the four DEN treated groups showed higher rates of hypermethylation and decreased mRNA levels of *p16*. This indicates that *p16 *hypermethylation induces *p16 *silencing in the rat liver cells, correlating with the development of liver cancer.

*C-myc *is an important regulator of various cellular processes, and has been shown to drive quiescent cells into the S phase in the absence of other mitogenic signals [5,7,12]. Diverse cellular functions of the *c-myc *oncogene are closely tied to its ability to either activate or repress gene transcription. Recently, a few studies have indicated that hypomethylation of the *c-myc *gene results in its over-expression in hepatocarcinogenesis, especially in the methyl-absent conditions [10,11].

As a methyl donor, betaine is proposed to play a role in homocysteine metabolism [36], and provides methyl groups for the synthesis of S-adenosylmethionine (SAM) [18,19,37]. The requirement of SAM for cellular metabolism normally exceeds what mammals obtain through their diets. Insufficiency in SAM can be prevented through the methionine cycle that metabolizes 5'-methyltetrahydrofolate (5'-methyl-THF) and betaine [38]. In the methionine cycle, both an increase in s-adenosyl-l-homocysteine (SAH) level and a decrease in the SAM:SAH ratio are known to reduce DNA methyltransferase activity and inhibit transmethylation reactions [18,19,37,39,42,43]. Betaine is effective at increasing the SAM:SAH ratio and supposed to maintain normal DNA methylation patterns [40].

In the present study, we have, however, found that betaine had no effect on methylation of *p16 *or *c-myc *by MSP and there were no differences in *c-myc *methylation among the five groups of rats. It may be necessary to use more sensitive methods to quantify methylation levels. It is also possible that betaine plays a key role in maintaining complete organism-wide methylation, not the methylation of a few, specific genes. This requires further investigations.

There are two re-methylation pathways utilized by betaine and 5'-methyl-THF, which are interrelated in the methionine cycle. The limitation of one pathway increases re-methylation *via *the other pathway [41]. Folic acid is the precursor of 5'-methyl-THF, and it also takes part in certain important procedures like the synthesis and reparation of DNA and RNA in addition to its effects on regulating DNA methylation. Having efficient methyl groups with three active methyls, betaine may reduce the consumption of folic acid in the methionine cycle, and it may play some part in maintaining DNA stability [42,43]. After metabolism, betaine molecules are degraded to N^5^, N^10^-methenyl-tetra-hydrofolic acid and N^5^, N^10^-methylene-tetra-hydrofolic acid, which can be reused in the synthesis of purine and thymine. These actions can regulate the mRNA expression.

We found *c-myc *over-expression in the four DEN treated groups, while the enhancement was inhibited by betaine in a dose-dependent manner. Meanwhile, betaine supplementation also enhanced the down-regulation of *p16 *induced by DEN in a dose-dependent manner. These results suggest that betaine regulates transcription of *c-myc *and *p16*, which leads to the stability of the two genes in the body and attenuate the carcinogenic effects of DEN.

## Conclusion

DEN induces disequilibrium in the lipid peroxidation and antioxidation systems of the rat liver, and alters mRNA expression levels of both *p16 *and *c-myc*. Betaine supplementation attenuates carcinogenic effects of DEN in rat livers and reverses DEN-induced changes in *p16 *and *c-myc *mRNA expression.

## Abbreviations

DEN: diethylinitrosamine; SAM: S-adenosylmethionine; ALT: alanine transaminase; AST: aspartate transaminase; ALP: alkaline phosphatase; GGT: γ-glutamyltransferase; MDA: malondialdehyde; SOD: superoxide dismutase; GSH: reduced glutathione; GST: glutathione S-transferase; T-AOC: total antioxidative capacity; MSP: Methylation-specific PCR; Q-PCR: Real time PCR; MBPs: methyl-binding proteins; SAH: s-adenosyl-l-homocysteine; 5'-methyl-THF: 5'-methyltetrahydrofolate.

## Competing interests

The authors declare that they have no competing interests.

## Authors' contributions

YPD carried out the animal studies and the methylation experiments, and drafted the manuscript. JSP performed data analysis and participated in the design of the study. AS carried out the immunoassays. ZHT carried out the realtime PCR experiments. WHL participated in the design and coordination of the study. HLZ conceived of the study and revised the manuscript. All authors read and approved the final manuscript.

## Pre-publication history

The pre-publication history for this paper can be accessed here:

http://www.biomedcentral.com/1471-2407/9/261/prepub

## References

[B1] Parkin DM Bray F Ferlay J Pisani P Global cancer statistics, 2002 CA Cancer J Clin 2005 55 2 74 108 10.3322/canjclin.55.2.74 15761078

[B2] Wang P Meng ZQ Chen Z Lin JH Ping B Wang LF Wang BH Liu LM Diagnostic value and complications of fine needle aspiration for primary liver cancer and its influence on the treatment outcome-a study based on 3011 patients in China Eur J Surg Oncol 2008 34 541 546 17764885 10.1016/j.ejso.2007.07.013

[B3] Sreepriya M Geetha B Chemopreventive effects of embelin and curcumin against N-nitrosodiethylamine/phenobarbital-induced hepatocarcinogenesis in Wistar rats Fitoterapia 2005 76 549 555 10.1016/j.fitote.2005.04.014 16009505

[B4] Sicklick JK Li YX Jayaraman A Kannangai R Qi Y Vivekanandan P Ludlow JW Owzar K Chen W Torbenson MS Dysregulation of the Hedgehog pathway in human hepatocarcinogenesis Carcinogenesis 2006 27 748 757 10.1093/carcin/bgi292 16339184

[B5] Mai S Mushinski JF c-Myc-induced genomic instability J Environ Pathol Toxicol Oncol 2003 22 179 199 10.1615/JEnvPathToxOncol.v22.i3.30 14529093

[B6] Filipski E Li XM Lévi F Disruption of circadian coordination and malignant growth Cancer Causes Control 2006 17 4 509 514 10.1007/s10552-005-9007-4 16596304

[B7] Beer S Komatsubara K Bellovin DI Kurobe M Sylvester K Felsher DW Hepatotoxin-induced changes in the adult murine liver promote MYC-induced tumorigenesis PLoS ONE 2008 3 e2493 10.1371/journal.pone.0002493 18560566 PMC2423614

[B8] Patel JH McMahon SB Targeting of Miz-1 is essential for Myc-mediated apoptosis J Biol Chem 2006 281 3283 3289 10.1074/jbc.M513038200 16352593

[B9] Cavin LG Wang F Factor VM Kaur S Venkatraman M Thorgeirsson SS Arsura M Transforming growth factor-alpha inhibits the intrinsic pathway of c-Myc-induced apoptosis through activation of nuclear factor-kappaB in murine hepatocellular carcinomas Mol Cancer Res 2005 3 403 412 10.1158/1541-7786.MCR-04-0186 16046551

[B10] Chen H Liu J Zhao CQ Diwan BA Merrick BA Waalkes MP Association of c-myc overexpression and hyperproliferation with arsenite-induced malignant transformation Toxicol Appl Pharmacol 2001 175 260 268 10.1006/taap.2001.9253 11559025

[B11] Tsujiuchi T Tsutsumi M Sasaki Y Hypomethylation of CpG sites and c-myc gene overexpression in hepatocellular carcinomas, but not hyperplastic nodules, induced by a choline-deficient L-amino acid-defined diet in rats Jpn J Cancer Res 1999 90 909 913 10551317 10.1111/j.1349-7006.1999.tb00834.x PMC5926157

[B12] Calvisi DF Conner EA Ladu S Lemmer ER Factor VM Thorgeirsson SS Activation of the canonical Wnt/beta-catenin pathway confers growth advantages in c-Myc/E2F1 transgenic mouse model of liver cancer J Hepatol 2005 42 842 849 10.1016/j.jhep.2005.01.029 15885355

[B13] Kim WY Sharpless NE The regulation of INK4/ARF in cancer and aging Cell 2006 127 265 275 10.1016/j.cell.2006.10.003 17055429

[B14] Ko E Kim Y Kim SJ Joh JW Song S Park CK Park J Kim DH Promoter hypermethylation of the p16 gene is associated with poor prognosis in recurrent early-stage hepatocellular carcinoma Cancer Epidemiol Biomarkers Prev 2008 17 2260 2267 10.1158/1055-9965.EPI-08-0236 18723830

[B15] Qin Y Liu JY Li B Sun ZL Sun ZF Association of low p16INK4a and p15INK4b mRNAs expression with their CpG islands methylation with human hepatocellular carcinogenesis World J Gastroenterol 2004 10 1276 1280 15112341 10.3748/wjg.v10.i9.1276 PMC4622765

[B16] Chu HJ Heo J Seo SB Kim GH Kang DH Song GA Cho M Yang US Detection of aberrant p16INK4A methylation in sera of patients with liver cirrhosis and hepatocellular carcinoma J Korean Med Sci 2004 19 83 86 14966347 10.3346/jkms.2004.19.1.83 PMC2822270

[B17] Björkhem-Bergman L Torndal UB Eken S Nyström C Capitanio A Larsen EH Björnstedt M Eriksson LC Selenium prevents tumor development in a rat model for chemical carcinogenesis Carcinogenesis 2005 26 125 131 10.1093/carcin/bgh290 15459019

[B18] Duong FH Christen V Filipowicz M Heim MH S-Adenosylmethionine and betaine correct hepatitis C virus induced inhibition of interferon signaling in vitro Hepatology 2006 43 796 806 10.1002/hep.21116 16557551

[B19] Kharbanda KK Rogers DD 2nd Mailliard ME Siford GL Barak AJ Beckenhauer HC Sorrell MF Tuma DJ Role of elevated S-adenosylhomocysteine in rat hepatocyte apoptosis: protection by betaine Biochem Pharmacol 2005 70 1883 1890 10.1016/j.bcp.2005.09.021 16253211

[B20] Craig SA Betaine in human nutrition Am J Clin Nutr 2004 80 539 549 15321791 10.1093/ajcn/80.3.539

[B21] Clow KA Treberg JR Brosnan ME Brosnan JT Elevated tissue betaine contents in developing rats are due to dietary betaine, not to synthesis J Nutr 2008 138 1641 1646 18716163 10.1093/jn/138.9.1641

[B22] Reeves PG Nielsen FH Fahey GC AIN-93 purified diets for laboratory rodents: final report of the American Institute of Nutrition ad hoc writing committee on the reformulation of the AIN-76A rodent diet J Nutr 1993 123 1939 1951 8229312 10.1093/jn/123.11.1939

[B23] Bagnyukova TV Tryndyak VP Montgomery B Churchwell MI Karpf AR James SR Muskhelishvili L Beland FA Pogribny IP Genetic and epigenetic changes in rat preneoplastic liver tissue induced by 2-acetylaminofluorene Carcinogenesis 2008 29 638 646 10.1093/carcin/bgm303 18204080

[B24] Zhao J Chen J Lu B Dong L Wang H Bi C Wu G Guo H Wu M Guo Y TIP30 induces apoptosis under oxidative stress through stabilization of p53 messenger RNA in human hepatocellular carcinoma Cancer Res 2008 68 4133 4141 10.1158/0008-5472.CAN-08-0432 18519672

[B25] Mishra P Kale RK Kar A Chemoprevention of mammary tumorigenesis and chemomodulation of the antioxidative enzymes and peroxidative damage in prepubertal Sprague Dawley rats by Biochanin A Mol Cell Biochem 2008 312 1 9 10.1007/s11010-008-9714-8 18273562

[B26] Ogborn MR Nitschmann E Bankovic-Calic N Buist R Peeling J Dietary betaine modifies hepatic metabolism but not renal injury in rat polycystic kidney disease Am J Physiol Gastrointest Liver Physiol 2000 279 G1162 1168 11093938 10.1152/ajpgi.2000.279.6.G1162

[B27] Lee MS Kim MS Park SY Kang CW Effects of betaine on ethanol-stimulated secretion of IGF-I and IGFBP-1 in rat primary hepatocytes: Involvement of p42/44 MAPK activation World J Gastroenterol 2006 12 1718 1722 16586540 10.3748/wjg.v12.i11.1718 PMC4124346

[B28] Erman F Balkan J Cevikbaş U Koçak-Toker N Uysal M Betaine or taurine administration prevents fibrosis and lipid peroxidation induced by rat liver by ethanol plus carbon tetrachloride intoxication Amino Acids 2004 27 199 205 10.1007/s00726-004-0105-5 15338317

[B29] Hayes KC Pronczuk A Cook MW Robbins MC Betaine in sub-acute and sub-chronic rat studies Food Chem Toxicol 2003 41 1685 1700 10.1016/S0278-6915(03)00196-0 14563394

[B30] Pradeep K Mohan CV Gobianand K Karthikeyan S Effect of Cassia fistula Linn. leaf extract on diethylnitrosamine induced hepatic injury in rats Chem Biol Interact 2007 167 12 18 10.1016/j.cbi.2006.12.011 17289008

[B31] Zhu JD The altered DNA methylation pattern and its implications in liver cancer Cell Res 2005 15 272 280 10.1038/sj.cr.7290296 15857582

[B32] Sansom OJ Maddison K Clarke AR Mechanisms of disease: methyl-binding domain proteins as potential therapeutic targets in cancer Nat Clin Pract Oncol 2007 4 305 315 10.1038/ncponc0812 17464338

[B33] Huang X Colgate KC Kolbanovskiy A Amin S Geacintov NE Conformational changes of a benzo[a]pyrene diol epoxide-N(2)-dG adduct induced by a 5'-flanking 5-methyl-substituted cytosine in a (Me)CG double-stranded oligonucleotide sequence context Chem Res Toxicol 2002 15 438 444 10.1021/tx015588h 11896693

[B34] Bai H Gu L Zhou J Deng D p16 hypermethylation during gastric carcinogenesis of Wistar rats by N-methyl-N'-nitro-N-nitrosoguanidine Mutat Res 2003 535 73 78 12547284 10.1016/s1383-5718(02)00288-7

[B35] Wong IH Lo YM Zhang J Liew CT Ng MH Wong N Lai PB Lau WY Hjelm NM Johnson PJ Detection of Aberrant p16 Methylation in the Plasma and Serum of Liver Cancer Patients Cancer Res 1999 59 71 73 9892188

[B36] Yagisawa M Okawa N Shigematsu N Nakata R Effects of intravenous betaine on methionine-loading-induced plasma homocysteine elevation in rats J Nutr Biochem 2004 15 666 671 10.1016/j.jnutbio.2004.05.004 15590270

[B37] Pogribny IP Ross SA Wise C Pogribna M Jones EA Tryndyak VP James SJ Dragan YP Poirier LA Irreversible global DNA hypomethylation as a key step in hepatocarcinogenesis induced by dietary methyl deficiency Mutat Res 2006 593 80 87 16144704 10.1016/j.mrfmmm.2005.06.028

[B38] Purohit V Abdelmalek MF Barve S Benevenga NJ Halsted CH Kaplowitz N Kharbanda KK Liu QY Lu SC McClain CJ Role of S-adenosylmethionine, folate, and betaine in the treatment of alcoholic liver disease: summary of a symposium Am J Clin Nutr 2007 86 14 24 17616758 10.1093/ajcn/86.1.14

[B39] Fang M Chen D Yang CS Dietary polyphenols may affect DNA methylation J Nutr 2007 137 Suppl 223S 228S 17182830 10.1093/jn/137.1.223S

[B40] Kharbanda KK Rogers DD 2nd Mailliard ME Siford GL Barak AJ Beckenhauer HC Sorrell MF Tuma DJ A comparison of the effects of betaine and S-adenosylmethionine on ethanol-induced changes in methionine metabolism and steatosis in rat hepatocytes J Nutr 2005 135 519 524 15735087 10.1093/jn/135.3.519

[B41] Melse-Boonstra A Holm PI Ueland PM Olthof M Clarke R Verhoef P Betaine concentration as a determinant of fasting total homocysteine concentrations and the effect of folic acid supplementation on betaine concentrations Am J Clin Nutr 2005 81 1378 1382 15941890 10.1093/ajcn/81.6.1378

[B42] Navarro-Perán E Cabezas-Herrera J Campo LS Rodríguez-López JN Effects of folate cycle disruption by the green tea polyphenol epigallocatechin-3-gallateInt J Biochem Cell Biol 2007 39 2215 2225 10.1016/j.biocel.2007.06.005 17683969

[B43] Radivoyevitch T Folate system correlations in DNA microarray data BMC Cancer 2005 5 95 10.1186/1471-2407-5-95 16080796 PMC1198223

